# Exceptionally high incidence of symptomatic grade 2–5 radiation pneumonitis after stereotactic radiation therapy for lung tumors

**DOI:** 10.1186/1748-717X-2-21

**Published:** 2007-06-07

**Authors:** Hideomi Yamashita, Keiichi Nakagawa, Naoki Nakamura, Hiroki Koyanagi, Masao Tago, Hiroshi Igaki, Kenshiro Shiraishi, Nakashi Sasano, Kuni Ohtomo

**Affiliations:** 1 Department of Radiology, University of Tokyo Hospital, Japan

## Abstract

**Background:**

To determine the usefulness of dose volume histogram (DVH) factors for predicting the occurrence of radiation pneumonitis (RP) after application of stereotactic radiation therapy (SRT) for lung tumors, DVH factors were measured before irradiation.

**Methods:**

From May 2004 to April 2006, 25 patients were treated with SRT at the University of Tokyo Hospital. Eighteen patients had primary lung cancer and seven had metastatic lung cancer. SRT was given in 6–7 fields with an isocenter dose of 48 Gy in four fractions over 5–8 days by linear accelerator.

**Results:**

Seven of the 25 patients suffered from RP of symptomatic grade 2–5 according to the NCI-CTC version 3.0. The overall incidence rate of RP grade2 or more was 29% at 18 months after completing SRT and three patients died from RP. RP occurred at significantly increased frequencies in patients with higher conformity index (CI) (p = 0.0394). Mean lung dose (MLD) showed a significant correlation with V_5_–V_20 _(irradiated lung volume) (p < 0.001) but showed no correlation with CI. RP did not statistically correlate with MLD. MLD had the strongest correlation with V_5_.

**Conclusion:**

Even in SRT, when large volumes of lung parenchyma are irradiated to such high doses as the minimum dose within planning target volume, the incidence of lung toxicity can become high.

## 1. Background

Since 1990, stereotactic radiotherapy (SRT) has been widely available for the treatment of intracranial lesions. Recently, the use of SRT has gradually been expanded to include the treatment of extra-cranial lesions. In particular, SRT has been demonstrated as a safe and effective modality in the treatment of primary and metastatic lung tumors [1]. Initial clinical results were favorable, and local control rates around 90% have been reported [1-9]. Since May 2004, we have employed SRT for body trunk tumors using a simple body cast system at the University of Tokyo Hospital.

Regarding normal tissue, the use of a single dose rather than a conventional fractionated dose may increase the risk of complications. However, few cases with severe toxicity have been reported [10].

A few patients undergoing high-dose SRT suffered from RP, which was treated by administration of steroids. The percentage of total lung volume receiving greater than or equal to 20 Gy (V_20_) was reported to be a useful factor for RP in conventional fractions [11]. The useful dose volume histogram (DVH) factors were examined for predicting the occurrence of RP after SRT for lung tumors.

## 2. Methods

### 2.1. Patients and tumor characteristics

From May 2004 to April 2006, 25 patients were treated with SRT using a stereotactic body cast system using a custom bed and low temperature thermoplastic material RAYCAST^® ^(ORFIT Industries, Wijnegem, Belgium) at the University of Tokyo Hospital. All patients enrolled in this study satisfied the following eligibility criteria: 1) solitary or double lung tumors; 2) tumor diameter < 40 mm; 3) no evidence of regional lymph node metastasis; 4) Karnofsky performance status scale ≧ 80% ; and 5) tumor not located adjacent to major bronchus, esophagus, spinal cord, or great vessels. Of the 25 patients, 16 had primary lung cancer, seven had metastatic lung cancer, and two had recurrent lung cancer. Ten patients were inoperable because of coexisting disease and one refused surgery. The primary lung cancers were staged as T1N0M0 in 15 and T2N0M0 in one. The primary sites of the metastases were the rectum, kidney, and ampulla of Vater in one each. A complete history was taken from all patients, and each received a physical examination, blood test, chest computed tomography (CT) scan, and whole-body positron emission tomography (PET) scan using FDG before treatment. Patient characteristics are summarized in Table 1.

**Table 1:** Details of patient characteristics

**No.**	**Age**	**Sex**	**Primary site**	**Subject**	**Histology of target lesion**	**Chronic Lung Disorder**	**Inoperable reason**	**K-PS (%)**	**s KL (U/ml)**	**s SP-D (ng/ml)**	**VC (L)**	**FEV1.0 (L)**
1	75	M	lung	primary	Adenoca	No	reject	90	wnl	wnl	4.07	2.81
2	83	M	lung	primary	Unknown	No	TAA/IHD	90	wnl	wnl	NA	NA
3	50	F	rectum	metastasis	Adenoca	post lobectomy	rectal ca.	90	wnl	wnl	3.40	2.66
4	77	M	lung	recurrence	SCLC	emphysema	SCLC-ED	90	wnl	wnl	NA	NA
5	75	M	lung	primary	Adenoca	No	nephrotic syndrome	80	wnl	wnl	NA	NA
6	60	M	lung	metastasis	Adenoca	post lobectomy	metastasis	90	743	wnl	2.61	0.59
7	79	M	lung	primary	SqCC	emphysema	colon ca./prostate ca.	90	wnl	wnl	1.75	1.26
8	79	M	ampulla of Vater	metastasis	Unknown	No	metastasis	80	wnl	wnl	NA	NA
9	69	M	lung	recurrence	Aenoca	post partial resection	recurrence	90	wnl	wnl	NA	NA
10	84	M	lung	primary	SqCC	No	TAA	70	wnl	wnl	1.74	0.85
11	81	M	lung	primary	Adenoca	No	M valve replacement	80	wnl	wnl	3.19	2.30
12	82	M	lung	primary	SqCC	No	prostate ca.	80	wn	wn	2.50	1.75
13	72	M	lung	metastasis	SqCC	No	metastasis	80	950	NA	2.76	2.13
14	80	M	lung	primary	Unknown	emphysema	HCC/colon ca.	80	NA	NA	NA	NA
15	80	M	kidney	metastasis	Unknown	No	Renal cell carcinoma	80	529	wnl	NA	NA
16	60	M	lung	metastasis	Carcinoma	IP	metastasis	80	852	NA	4.01	3.24
17	77	M	lung	primary	NSCLC	IP	IP	80	1590	NA	3.05	1.59
18	68	M	lung	primary	Adenoca	COPD	COPD	70	NA	NA	NA	NA
19	79	M	lung	primary	SqCC	emphysema	AAA	90	520	NA	NA	NA
20	64	F	lung	primary	Adenoca	No	CRF/IHD	90	wnl	wnl	2.04	1.56
21	76	F	lung	primary	SCLC	No	bladder ca./breast ca.	90	wnl	wnl	2.17	1.59
22	77	M	lung	primary	SqCC	No	diabetic nephropathy	80	wnl	wnl	NA	NA
23	78	M	lung	primary	NSCLC	IP	IP	80	wnl	127	NA	NA
24	62	M	colon	metastasis	Unknown	No	colon ca.	90	wnl	wnl	3.69	2.87
25	78	F	lung	primary	Carcinoma	IP/post lobectomy	post lobectomy	90	wnl	wnl	1.54	0.99

									(0–500)	(0–110)		

AAA: abdominal aortic aneurysm, Adenoca: adenocarcinoma, ca.: cancer, COPD: chronic obstructive pulmonary disea

ED: extended disease, FEV: forced expiratory volume, HCC: hepatocellular carcinoma, IHD: ischemic heart disease, IP?

K-PS: karnofsky performance status scale, M valve: mitral valve, NA: not available, s: serum, TAA: thoracic aortic ane

RP: radiation pneumonitis, SCLC:small cell lung cancer, SP-D: surfactant protein-D, SqCC: squamous cell carcinoma,

In our clinical cases, five could not be histologically confirmed because the patients could not tolerate CT-guided biopsy and transbronchoscopic lung biopsy (TBLB). In these patients, the tumor diagnosis was confirmed clinically by a growing tumor on repeated CT scans and by exclusion of another primary tumor by clinical staging. None of the patients received concurrent chemotherapy with SRT. Additionally, no chemotherapy, which might affect the RP rates, was given prior to or immediately after SRT (until two months).

### 2.2. Planning procedure and treatment

The patient was positioned in a supine position on a custom bed. A body cast was made to broadly cover the chest to the abdomen during shallow respiration, and attached rigidly to the sidewall of the base plate.

The CT slice thickness and pitch were 1 mm each in the area of the tumor, and 5 mm each in the other areas. Each CT slice was scanned with an acquisition time of four seconds to include the whole phase of one respiratory cycle. A series of CT images, therefore, included the tumor and its respiratory motion. The axial CT images were transferred to a 3-dimension RT treatment-planning machine (Pinnacle^3^, New Version 7.4i, Philips). Treatment planning was performed using the 3D RTP machine. The target volume corresponded to the internal target volume (ITV) in Japan Clinical Oncology Group (JCOG) 0403 phase II protocol [12]. The CT images already included the internal motion because long scan time (four seconds) CT under free breathing (what is called, "slow" CT scan) was used [13,14]. Spicula formation and pleural indentation were included within the ITV. The setup margin (SM) between ITV and the planning target volume (PTV) was 5 mm in all directions. Additionally, there was additional 5 mm leaf margin to PTV, according to JCOG0403 protocol, in order to make the dose distribution within the PTV more homogeneous. Two to 4 multi-leaf-collimator (MLC)-shaped non-coplanar static ports of 6-MV X-rays were selected to decrease mean lung dose (MLD), V_20_, and V_15 _to below 18.0 Gy, 20%, and 25%, respectively, according to JCOG0403 protocol, although such numbers as V20 < 20% and V15 < 25% were valid for fractionation doses of about 2 Gy. We used no pairs of parallel opposing fields. The target reference point dose was defined at the isocenter of the beam. The collapsed cone (CC) convolution method was used as the dose calculation, in which the range of Compton electrons was better taken into account. In short, the convolution describes radiation interactions including charged particle transport, and calculates dose derived from CT density and patient set up information. The collapsed cone convolution method uses an analytical kernel represented by a set of cones, the energy deposited in which is collapsed onto a line (hence the name). The method is used to reduce computation time. In practice, the method utilizes a lattice of rays, such that each voxel is crossed by one ray corresponding to each cone axis. The primary beams were calculated heterogeneously and the scatter beams homogeneously as dose computation parameters. SRT was given with a central dose of 48 Gy in four fractions over 5–8 days in 6–7 fields by linear accelerator (SRL6000, Mitsubishi Electric Co., Tokyo) excluding two cases. Two patients (case no. 14 and 19) received 48 Gy in more than 4 fractionations (6 and 8 fractionations, respectively) (Table 2) since the tumor located in the hilar (central) region. As to the peripheral dose of the PTV, we checked that 95% PTV volumes coverage dose (D95) was over 90% of the central dose. CT verification of the target isocenter was performed to ensure the correct target position and sufficient reproducibility of suppressing breathing mobility before each treatment session.

**Table 2:** DVH characteristics in treatment planning.

**No.**	**Tumor location**	**Isocenter Dose**	**BED_10 _(Gy)**	**Beam**	**Co-pulanar**	**Collimators (mm)**	**Field size (mm^2^)**	**V_20 _(%)**	**V_40 _(%)**	**V_45 _(%)**	**MLD (cGy)**	**D95 (cGy)**	**HI (%)**	**CI (%)**
1	peripheral	48Gy/4f	105.6	6	2	67 × 74	4958	5.0	2.0	1.0	206	4408	126	171
2	peripheral	48Gy/4f	105.6	6	2	40 × 61	2440	5.0	2.0	1.0	488	4547	128	219
3	peripheral	48Gy/4f	105.6	6	2	30 × 31	930	1.0	0.5	0.3	172	4462	120	202
4	peripheral	48Gy/4f	105.6	6	2	60 × 46	2760	7.0	3.0	2.0	445	4325	128	147
5	peripheral	48Gy/4f	105.6	6	2	48 × 63	3024	3.0	2.0	1.0	298	4443	117	157
6	peripheral	48Gy/4f	105.6	6	2	67 × 67	4489	8.0	3.0	1.0	406	4435	123	197
7	peripheral	48Gy/4f	105.6	6	2	49 × 57	2793	8.0	2.0	1.0	510	4432	118	187
8	peripheral	48Gy/4f	105.6	6	2	45 × 51	2295	3.0	1.0	0.5	259	4468	125	182
9	peripheral	48Gy/4f	105.6	6	2	55 × 60	3300	7.0	2.0	1.0	404	4515	118	168
10	peripheral	48Gy/4f	105.6	6	2	59 × 68	4012	9.0	2.0	1.0	573	4511	122	170
11	peripheral	48Gy/4f	105.6	6	2	69 × 68	4692	7.0	2.9	2.0	404	4380	126	204
12	peripheral	48Gy/4f	105.6	7	2	79 × 97	7663	9.0	6.1	5.2	579	4355	134	169
13	rt perihilar/central	48Gy/4f	105.6	6	2	51 × 51	2601	7.0	2.7	1.9	585	4633	112	322
	lt perihilar/central	48Gy/4f	105.6	6	2	49 × 57	2793	6.0	2.6	1.9	353	4629	110	257
14	perihilar/central	48Gy/8f	76.8	6	2	45 × 63	2835	7.0	1.0	0.5	568	4557	124	184
15	peripheral	48Gy/4f	105.6	6	2	40 × 42	1680	5.0	2.0	1.0	313	4617	109	282
16	peripheral	48Gy/4f	105.6	6	2	70 × 54	3780	10.0	5.0	3.0	791	4500	126	173
17	peripheral	48Gy/4f	105.6	6	2	48 × 62	2976	6.0	1.0	0.5	426	4405	121	310
18	peripheral	48Gy/4f	105.6	6	2	55 × 53	2915	4.0	1.0	0.5	291	4780	115	148
19	perihilar/central	48Gy/6f	86.4	6	2	59 × 59	3481	11.0	3.0	1.0	541	4835	139	170
20	peripheral	48Gy/4f	105.6	7	4	49 × 46	2254	11.0	1.0	0.5	321	4851	112	164
21	peripheral	48Gy/4f	105.6	6	2	50 × 56	2800	6.0	1.0	0.5	426	4602	118	192
22	peripheral	48Gy/4f	105.6	6	2	55 × 57	3135	7.0	2.0	1.0	440	4890	119	175
23	peripheral	48Gy/4f	105.6	7	4	60 × 58	3480	8.0	2.0	1.0	422	4585	112	130
24	peripheral	48Gy/4f	105.6	6	2	35 × 34	1190	2.0	0	0	230	4468	117	173
25	peripheral	48Gy/4f	105.6	6	2	32 × 40	1280	4.0	0.5	0	353	4591	107	153

BED: biologically effective doses, CI: conformity index, f: fractions, HI: homogeneity index, MLD: mean lung dose, Vx: irradiated lung volume more than × Gy

### 2.3. Evaluation of clinical outcome

After completing SRT, chest x-ray films and serial chest CT scans were checked for all cases to evaluate treatment outcomes at 2, 4, 6, 9, 12, 18, and 24 months after completion. Routine blood test results were also examined in all cases at the same time. Lactate dehydrogenase (LDH) and serum Krebs von den Lungen-6 (KL-6) were also collected at the same time as a serum marker of RP. The local tumor response was evaluated using the Response Evaluation Criteria in Solid Tumors Group [15]. Tumor response was assessed by follow-up chest radiography and CT scan. In accordance with WHO criteria, tumor response was defined as complete if all abnormalities that were anatomically related to the tumor disappeared after treatment, and defined as partial if the maximum size of these abnormalities decreased by ≧ 50%. Toxicities were evaluated using the National Cancer Institute-Common Toxicity Criteria (NCI-CTC) version 3.0. The toxicity data was collected retrospectively from the patient files. The following grading system was assigned to the RP: Grade 1, asymptomatic (radiographic findings only); Grade 2, symptomatic and not interfering with activities of daily living (ADL); Grade 3, symptomatic and interfering with ADL or O_2 _indicated; Grade 4, life-threatening (ventilatory support indicated), and Grade 5, death.

Maximum dose, minimum dose, D95, field size, and homogeneity index (HI) were evaluated (Table 2). HI was defined as the ratio of maximum dose to minimum dose. In our institution, HI must be below 1.40 in order to keep the dose within the PTV more homogeneous. In analyzing the dose to the lung, the V_5_-V_20_, MLD, and conformity index (CI) were evaluated (Table 2). V_5_-V_50 _and MLD was calculated for both lungs. The lung volume minus the PTV (PTV excluded) was used as the volume of lung parenchyma. In this study, CI was defined as the ratio of treated volume (TV) (the definition of TV was the volume covered by minimum dose within PTV) to PTV (i.e. CI = TV/PTV) according to JCOG0403 protocol, although this concept might be old and be used hardly. This definition of the CI is the opposite comparing with the CI defined by Knoos *et al. *(CI = PTV/TV) [16]. The higher the CI values obtained indicated that the areas irradiated were less conformal. Three patients had lesions located in the hilar/central tumor region according to Timmerman *et al. *[10].

### 2.4. Statistical analysis

CI and MLD between RP positive and negative were compared using an unpaired multiple *t*-tests. Statistical significant was defined as *p *value of <0.05.

## 3. Results

The patients ranged in age from 50 to 84 years with a median of 77 years (73.8 ± 8.6 years). Female to male ratio was 4:21. The volumes irradiated over 5, 7, 10, 13, 15, 20, 30, 35, 40, 45, 50 Gy were designated as V_5_, V_7_, V_10_, V_13_, V_15_, V_20_, V_30_, V_35_, V_40_, V_45_, V_50 _respectively. Nine patients had chronic lung disorders, and four were in a postoperative state. Four patients had emphysema, three had interstitial pneumonia (IP), and one had chronic obstructive pulmonary disease (COPD). The length of follow-up ranged from 10 to 28 months with a median of 17 months (16.1 ± 7.1 months). During the follow-up period, only two tumors showed local regrowth in the meaning of local control (Table 3). The overall radiation treatment-time was five or 6 days in all cases excluding a single patient and the single patient was 8 days. The absolute volumes for every patient: ITV, PTV, the volume enclosed by the 48Gy total-isodose, the 24Gy-isodose-volume were shown in Table 4.

**Table 3:** Treatment results and RP grading

**No.**	**Follow up (Months)**	**Dead or alive (cause of death)**	**Local control**	**Control out of field**	**RP grading**
1	16	dead (primary)	PD	PD	G0
2	19	dead (aging)	PR	control	G0
3	20	alive	PR	PD	G1
4	19	alive	PR	control	G1
5	19	alive	CR	control	G1
6	16	alive	PD	PD	G1
7	15	alive	CR	control	G1
8	10	dead (primary)	PR	control	G0
9	14	alive	CR	control	G0
10	4	dead (aging)	PR	control	G0
11	10	alive	PR	control	G2 (2Mo)
12	11	alive	PR	control	G1
13	4	dead (RP)	CR	control	G5 (3Mo)
14	11	alive	CR	control	G2 (5Mo)
15	10	alive	PR	control	G1
16	7	dead (RP)	CR	PD	G5 (6Mo)
17	9	alive	CR	control	G3 (6Mo)
18	9	alive	CR	control	G4 (9Mo)
19	9	alive	PR	control	G1
20	8	dead (primary)	CR	control	G1
21	8	alive	CR	control	G0
22	6	dead (RP)	CR	control	G5 (5Mo)
23	7	alive	PR	control	G1
24	3	alive	PR	control	G0
25	2	alive	NE	NE	G0

CR: complete response, NE: not evaluate, PD: progressive disease, PR: partial response, RP: radiation pneumonitis, SD: stable disease

**Table 4:** The absolute volumes for every patient: ITV, PTV, the volume enclosed by the 48Gy total-isodose, the 24Gy-isodose-volume

**Case**	**ITV (cm^3^)**	**PTV (cm^3^)**	**V48 (cm^3^)**	**V24 (cm^3^)**
1	13.9	66.8	54.4	284
2	10.1	40.1	25.9	108
3	1.0	7.5	0.0	30
4	9.6	34.9	6.7	141
5	11.4	45.2	0.3	85
6	34.2	85.1	22.8	166
7	17.2	51.0	3.0	135
8	9.7	33.7	10.5	57
9	16.4	54.4	8.1	175
10	30.8	81.9	25.3	258
11	30.0	79.1	37.5	212
12	126.9	239.4	98.4	263
13 rt	5.0	20.5	16.5	123
lt	6.4	26.4	15.6	114
14	15.5	47.5	20.1	147
15	5.0	10.2	3.4	109
16	49.9	120.9	46.7	303
17	5.1	29.4	6.4	128
18	13.2	42.5	0.6	247
19	36.2	85.0	6.8	238
20	8.4	29.0	2.1	81
21	9.0	29.6	1.7	103
22	18.5	56.5	1.7	119
23	17.3	50.8	1.8	153
24	1.8	10.6	2.6	39
25	1.7	10.5	0.4	36

**Figure 1:**
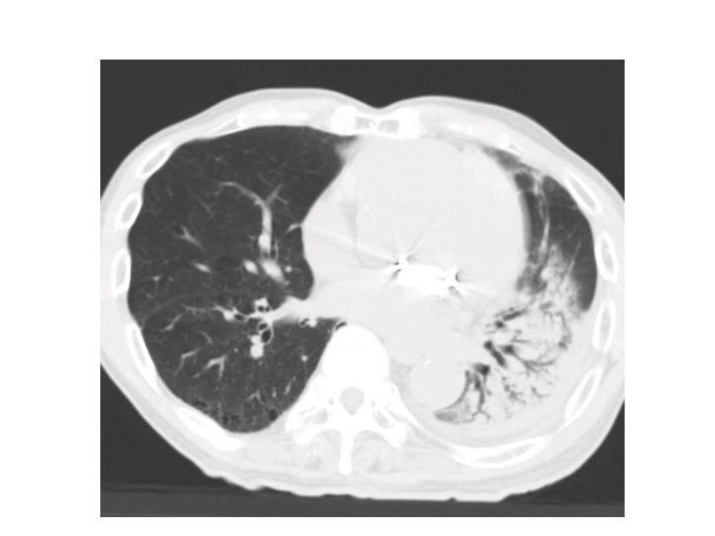
Computed tomography (CT) image of radiation pneumonitis (RP) (patient No. 11).

**Figure 2:**
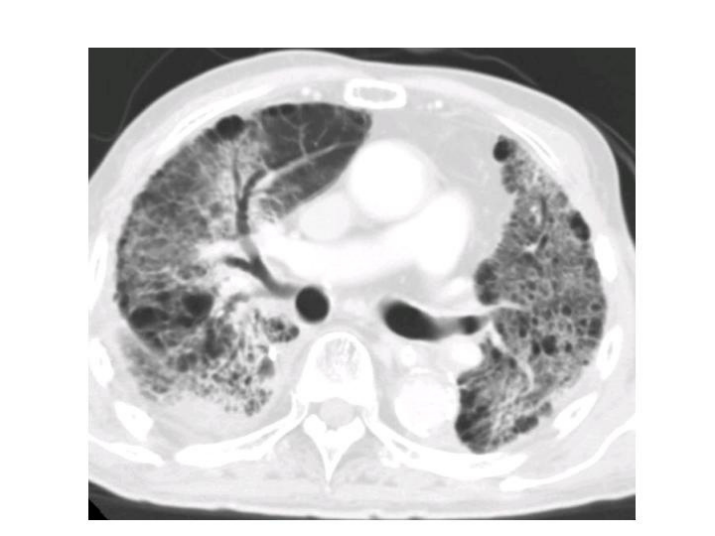
CT image of RP (patient No. 13).

**Figure 3:**
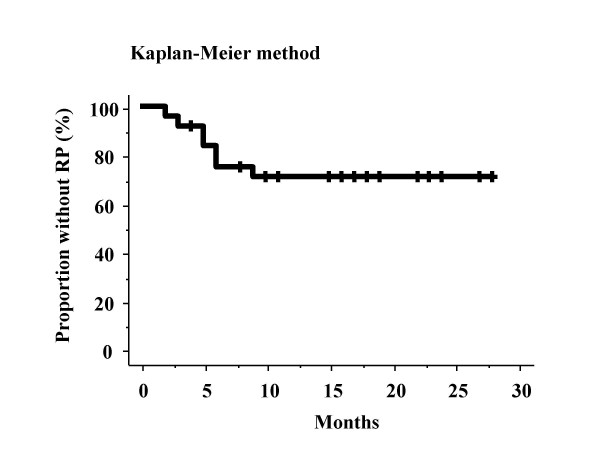
Kaplan-Meier plot of time from treatment until RP grade2 to 5. There were seven patients: one had RP at 2 months, one at 3 months, one at 9 months, two at 5 months, and two at 6 months.

Seven out of the 25 patients suffered from RP of grade 2 or more in the NCI-CTC version 3.0. All patients with RP had a cough, continuous fevers, severe dyspnea, and showed infiltrative changes in both irradiated and non-irradiated areas on chest CT (Figures 1 and 2). Three patients out of 25 treated with SRT died from a fatal RP. There were seven patients: one had RP at 2 months, one at 3 months, one at 9 months, two at 5 months, and two at 6 months. In all of the seven patients, pneumonitis spread out beyond the PTV. The overall incidence rate of RP grade 2 or more determined by the Kaplan-Meier method was 29.2% at 18 months after completing SRT (Figure 3). Various clinical as well as therapeutic factors were analyzed for their possible relationships to the incidence of RP (Table 2). There were no significant relations between the incidence of RP and with or without co-morbidity lung disease (χ^2 ^test: *p *= 0.9400). Only two cases (22%) developed RP out of nine patients with co-morbidity lung disease. In all of the 25 patients, LDH levels remained normal during the follow-up period. Three of the seven patients with RP had high values of serum KL-6 before SRT, and the other four had normal serum KL-6 level. Additionally, RP had been observed in three patients who had high levels of serum KL-6 before SRT.

**Figure 4:**
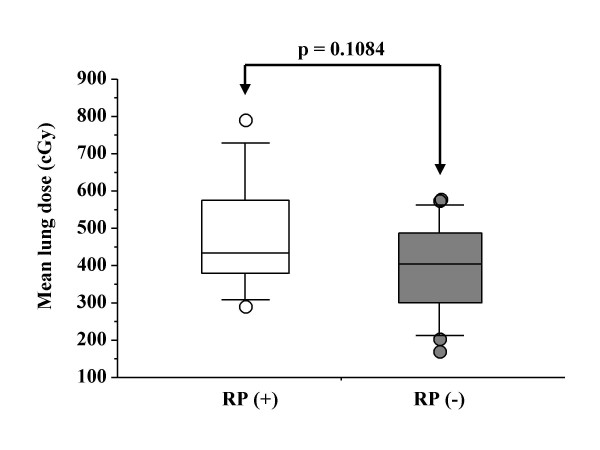
The correlation comparing the occurrence of RP grade 2 or more with MLD.

**Figure 5:**
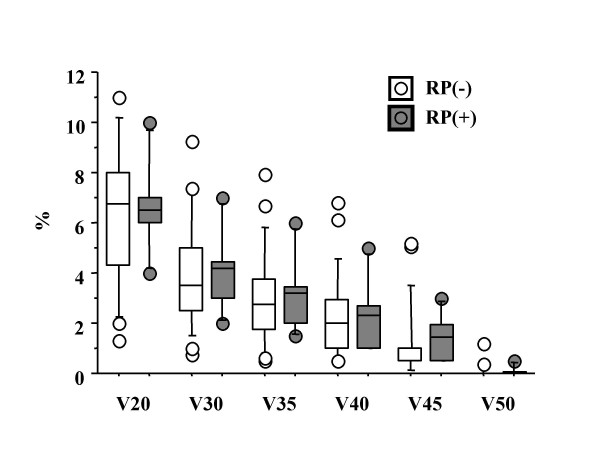
The correlation comparing the occurrence of RP grade 2 or more with V_20_-V_50_.

**Figure 6:**
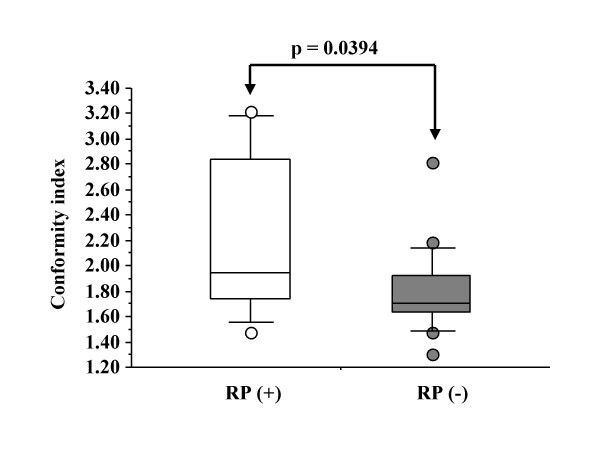
The correlation comparing the occurrence of RP grade 2 or more with CI.

The high value of CI showed a significant correlation with the occurrence of RP, while MLD (Figure 4), field size, PTV volume, and V_5_, V_7_, V_10_, V_13_, and V_15 _(p value according to unpaired *t*-test was 0.1966, 0.1658, 0.2351, 0.3831, and 0.3963, respectively) showed no correlations with RP. Additionally, V_20_, V_30_, V_35_, V_40_, V_45_, and V_50 _showed no significant correlations with the incidence of RP, either (p value was 0.6768, 0.8369, 0.8318, 0.8044, 0.7544, and 0.9218, respectively) (Figure 5). Even when the volumes V_5_-V_50 _were given in absolute units (cm^3^) for the lung parenchyma (PTV excluded), there were no significant correlations between V_5_–V_50 _and the incidence of RP (Table 5). The patients with RP had a mean CI of 222–66%, while the mean for patients without RP was 180–33% (*p *= 0.0394) (Figure 6). There was no significant correlation between both the ITV and PTV volume and the incidence of RP (*p *= 0.7415 and *p *= 0.7675, respectively).

**Table 5:** The correlation comparing the occurrence of RP with V5-V50

		**V5**	**V7**	**V10**	**V13**	**V15**	**V20**	**V30**	**V35**	**V40**	**V45**	**V50**
*p*	value RP	0.2500	0.2422	0.3208	0.2742	0.2717	0.4063	0.5858	0.7557	0.8220	0.9307	0.4780
	with	744 ± 134	631 ± 117	495 ± 95	368 ± 70	307 ± 56	210 ± 39	124 ± 24	96 ± 18	75 ± 15	48 ± 11	1 ± 1
	without	604 ± 52	504 ± 47	400 ± 43	290 ± 32	244 ± 26	174 ± 20	108 ± 14	88 ± 13	70 ± 11	47 ± 9	4 ± 2

mean ± SD (cm^3^)

CI showed no significant correlations with V_5_-V_20 _and MLD. CI correlated significantly with the ITV (both *t*-test and χ^2 ^test: *p *< 0.0001).

No patient had NCI-CTC Grade 3 or 4 toxicities such as fatigue, dermatitis associated with radiation, dysphagia, esophagitis, and pain in chest wall.

## 4. Discussion

Although extracranial stereotactic irradiation is an emerging treatment modality utilized by an increasing number of institutions in this field [1-4], only a few institutions have published their clinical results. SRT is accepted as a treatment method in medically inoperable non-small cell lung cancer or in patients who refused surgery. Promising results have been reported for this treatment method, with high local control rates and low incidence of complications [7,17-21]. A multi-institutional prospective trial (JCOG 0403) is currently in progress in Japan. This paper describes the experience of treating 25 patients with small (< 4 cm) lung tumors with four fractions of 12Gy. An unusually high rate of severe (grade 3 or more) RP (20%) and mortality (12%) was noticed and we are searching for reasons to explain these results, because we notice that these rates are far beyond other reported series. In this study, since the clinical data is collected retrospectively, the data is biased and there is a lack of information. Especially the lung function data of 11 patients (44%) are missing.

In our study, some of the patients started to suffer from "pneumonitis" almost 12 months after radiotherapy. These patients suffered from lung fibrosis plus pneumonia. RP is generally seen within 3 months of radiation and, in contrast, radiation fibrosis, which is thought to represent scar/fibrotic lung tissue, is usually a "late effect" seen >3 months after radiation. These may be difficult to distinguish from each other. RP is a sub-acute (weeks to months from treatment) inflammation of the end bronchioles and alveoli. The clinical picture may be very similar to acute bacterial pneumonia with fatigue, fever, shortness of breath, non-productive cough, and a pulmonary infiltrate on chest x-ray. The infiltrate on chest x-ray should include the area treated to high dose, but may extend outside of these regions. The infiltrates may be characteristically "geometric" corresponding to the radiation portal, but may also be ill defined.

CI may be a useful DVH factor for predicting the occurrence of RP after SRT for lung tumors. Although the CI was first proposed in 1993 by the Radiation Therapy Oncology Group (RTOG) and described in Report 62 of the International Commission on Radiation Units and Measurements (ICRU), it has not been included in routine practice [16,22-25]. The CI is a measure of how well the volume of a radiosurgical dose distribution conforms to the size and shape of a target volume, and is a complementary tool for scoring a given plan or for evaluating different treatment plans for the same patient. The radiation CI gives a consistent method for quantifying the degree of conformity based on iso-dose surfaces and volumes. Care during interpretation of radiation CI must always be taken, since small changes in the minimum dose can dramatically change the treated volume [16]. With the growth of conformal radiotherapy, the CI may play an important role in the future. However, this role has not yet been defined, probably because the value of conformal radiotherapy is just beginning to be demonstrated in terms of prevention of adverse effects and tumor control [26-29]. In our study, there was a significant association between CI with RP rate (*p *= 0.0394). A higher CI is less conformal. Figure 6 appears to say that the CI should be less than 2.00 since the most patients (15/18 cases) without RP were covered. This is a reflection of the number of beams and the spreading out of the prescribed dose. It is recommended that efforts be directed to reduce CI (= TV/PTV) in treatment planning. For that purpose, the minimum irradiation dose within PTV should be raised to reduce the TV. CI is generally used as a criterion to evaluate treatment plan. It has no relation with the volume of the irradiated lung. From a radiotherapeutic/-biological point of view, it is not likely that CI has a true predictive value for development of RP. CI is related to volume receiving very high radiation dose (90 % of prescribed dose). Lung tissue is vulnerable even to low dose. Therefore parameters related to volumes receiving low doses (i.e. V_10 _or MLD) are much more likely to correlate with toxicity. As the cases numbers were small, the co-relationship of CI and PR possibly may be coincident.

In our study, statistical analysis did not show significant association between MLD and RP rate, which were different from results of lung toxicity from conventional fractionation [11,30,31]. In our study, CI had no significant correlation with MLD. MLD was not a useful factor for predicting the occurrence of RP. V_5 _rather than V_7_, V_10_, V_13_, V_15_, and V_20 _had the strongest correlation with MLD, although in our study neither V_5 _nor MLD was a useful factor for predicting RP.

In a similar study by Paludan *et al. *[32] reporting dose-volume related parameters in a similar number of patients (N = 28), no relationship between DVH parameters and changes in dyspnea was found. They found that deterioration of lung function was more likely related to the patient co-morbidity (COPD) than to dose-volume related parameters. However, in the present analysis, there were no significant relations between the incidence of RP and with or without co-morbidity lung diseases.

The levels of KL-6 [17,33-35] and LDH are reported to be sensitive markers of RP, but in our study, both markers were not very sensitive. A few patients undergoing single high-dose SRT suffered from radiation pneumonitis, which was treated by administration of steroids. It is known that intense radiation changes and fibrosis without symptoms (Grade 1) will be found in the majority of patients after hypo-fractionated SRT. In addition, pneumonias develop regularly in these medically inoperable patients, and the combination of these can easily mislead to a diagnosis of RP. Misclassification in such a small number of patients will lead to a huge overestimation of the real incidence. In particular the fact that some of the patients already suffered from IP may have obscured the occurrence of RP. E.g. Figure 2 is at "best" a patient suffering from bronchiolitis obliterans with organizing pneumonia (BOOP), with the bilateral infiltrates.

It is debatable whether V_20 _can be applied to SRT in the same way as it is applied to conventional radiotherapy [11,36]. Our >20 Gy irradiated volume of the whole lung was 1.0–9.0% (average 4.83%), which was markedly smaller than that reported by Graham *et al. *[11]. In a previous study using whole-body irradiation, Wara *et al. *[37] demonstrated that eight Gy is the tolerance dose in the lung in single fractional irradiation. V_20 _was defined for standard fractionation. Biologically equivalent dose (BED) would be about 6.7 Gy (α/β = 3) with 12 Gy per fractionation. Thus, V_5 _and V_7 _would be important factor.

Many studies [7,18-20,38] have reported no patients who showed RP of Grade 3 or more in lung SRT. Additionally, only low incident rate of grade 2 RP (2.4% [20], 3% [21], 5.4% [18], and 7.2% [39]) was reported. Hara *et al. *[17] at the International Medical Center of Japan reported that 3 of the 16 patients (19%) experienced RP of Grade 3 severity with SRT of 20–35 Gy in a single fraction. Belderbos *et al. *[39] suggested additional reductions of the security margins for PTV definition and introduction of inhomogeneous dose distributions within the PTV. Compared with these reports, the occurrence rate of RP was much higher in our institution. As for its cause, we submit that many patients in our study had poor respiratory function, many patients were judged as inoperable because of IP, and some cases had recurrent lung tumors after surgery. If the relative gantry angles and the number of beams were arranged more properly, the CI ratio would be made lower, since their factors probably are directly related to the CI. Additionally it is essential to use small fields. We set the leaves at 5 mm outside the PTV in order to make the dose distribution within the PTV more homogeneous. This may be the reason why we got so unacceptably high CI. We might have had to set the leaves at the margin of the PTV according to the ongoing Radiation Therapy Oncology Group protocols. There must be something wrong with either the way targets are irradiated. Clinical target volume including spicula formation (= ITV) + 5 mm ITV-PTV margin + 5 mm PTV-leaf margins might have been unnecessary large margins. However, our PTV (53.4 ± 47.0 cm^3^, median: 43.8 cm^3^) was almost equal to the PTV reported by Fritz *et al. *[38] (median: 45.0 cm^3^) without any symptomatic RP. It appears that in this study large volumes of lung parenchyma were irradiated to such high doses as the minimum dose within planning target volume (= high the TV and high CI value), which may explain the high incidence of lung toxicity.

Timmerman *et al. *[10] recently published a paper reporting of a high incidence of RP after SRT. They found an unacceptable high rate, if the tumor was located more centrally. In our study, this tendency was not seen (only one out of patients with severe RP had a central tumor).

Hope *et al. *[40] found that RP is correlated to the volume of the high dose region. These data (the value of CI and the incidence of RP had the strongest correlation) may support another hypothesis that RP probably has associations with high dose regions rather than with low dose regions (V_5_-V_20_). However, in our study, V_30_, V_35_, V_40_, V_45_, and V_50 _showed no significant correlations with the incidence of RP, either. It may be no wonder that the CI does not show a relation with V_30_-V_50_, because the V_30_-V_50 _depends on the absolute volume of the PTV, not on the CI. Only the treatment technique will show such correlation.

The use of multiple non-coplanar static ports achieved homogeneous target dose distributions and avoided high doses to normal tissues, despite the limitation of the beam arrangement from the use of the body frame and couch structure.

## 5. Conclusion

In our institution, exceptionally high incidence of Grade 3–5 radiation pneumonitis after SRT for lung tumors was seen. Even in SRT, when large volumes of lung parenchyma are irradiated to such high doses as the minimum dose within planning target volume, the incidence of lung toxicity can become high. Further observations of the radiation changes in the lung after SRT are needed.

## Competing interests

The author(s) declare that they have no competing interests.

## Authors' contributions

• HY conducted follow-up examinations and contributed to data analysis and drafting the manuscript.

• KN oversaw the administration of radiation therapy to the patients, conducted follow-up.

• NN oversaw the administration of radiation therapy to the patients, conducted follow-up.

• HK contributed to data analysis and drafting the manuscript.

• MT oversaw the administration of radiation therapy to the patients, conducted follow-up.

• IH performed assessments of patients.

• KS performed assessments of patients.

• NS performed assessments of patients.

• KO contributed to drafting the manuscript.

All authors read and approved the final manuscript.
